# Comorbid Diseases Interact with Breast Cancer to Affect Mortality in the First Year after Diagnosis—A Danish Nationwide Matched Cohort Study

**DOI:** 10.1371/journal.pone.0076013

**Published:** 2013-10-09

**Authors:** Anne Gulbech Ording, Jens Peter Garne, Petra Mariann Witt Nyström, Trine Frøslev, Henrik Toft Sørensen, Timothy L. Lash

**Affiliations:** 1 Department of Clinical Epidemiology, Aarhus University Hospital, Aarhus, Denmark; 2 Breast Clinic, Aalborg Hospital, Aalborg University Hospital, Aalborg, Denmark; 3 Department of Oncology, Uppsala University Hospital, Uppsala, Sweden; 4 Department of Epidemiology, Rollins School of Public Health, Emory University, Atlanta, Georgia, United States of America; Florida International University, United States of America

## Abstract

**Background:**

Survival of breast cancer patients with comorbidity, compared to those without comorbidity, has been well characterized. The interaction between comorbid diseases and breast cancer, however, has not been well-studied.

**Methods:**

From Danish nationwide medical registries, we identified all breast cancer patients between 45 and 85 years of age diagnosed from 1994 to 2008. Women without breast cancer were matched to the breast cancer patients on specific comorbid diseases included in the Charlson comorbidity Index (CCI). Interaction contrasts were calculated as a measure of synergistic effect on mortality between comorbidity and breast cancer.

**Results:**

The study included 47,904 breast cancer patients and 237,938 matched comparison women. In the first year, the strongest interaction between comorbidity and breast cancer was observed in breast cancer patients with a CCI score of ≥4, which accounted for 29 deaths per 1000 person-years. Among individual comorbidities, dementia interacted strongly with breast cancer and accounted for 148 deaths per 1000 person-years within one year of follow-up. There was little interaction between comorbidity and breast cancer during one to five years of follow-up.

**Conclusions:**

There was substantial interaction between comorbid diseases and breast cancer, affecting mortality. Successful treatment of the comorbid diseases or the breast cancer can delay mortality caused by this interaction in breast cancer patients.

## Introduction

Breast cancer patients with comorbidities have poorer survival than breast cancer patients without comorbidity [1-5]. Few studies have compared mortality in breast cancer patients with coexisiting diseases to mortality in a comparable cohort of women free of breast cancer. One study provided evidence of statistical interaction between breast cancer and the Charlson Comorbidity Index (CCI [6]) score at the time of breast cancer diagnosis, but this study was hospital-based, only included 5,663 older patients, and did not study specific comorbidities [7]. Whether the survival difference is due to only the comorbidity or to an interaction between the comorbidity and breast cancer diagnosis is therefore not known. Such an interaction may have implications for disease treatment.

To resolve these limitations, we estimated the difference between the overall mortality rate and the expected mortality rate, given the baseline mortality rate, the effect of breast cancer on the mortality rate, and the effect of comorbidity on the mortality rate. We hypothesized *a priori* that the interaction may depend on the specific comorbid disease(s), and that the interaction may be different in the first year after breast cancer diagnosis than in subsequent years, since mortality in the first year is more likely affected by delayed breast cancer diagnosis and by treatment and toxicities.

## Methods

This nationwide study included a cohort of Danish breast cancer patients aged 45 to 85 years who were diagnosed between 1994 and 2008, and a comparison cohort of women without breast cancer matched to the breast cancer patients on specific diseases included in the CCI [6]. The population of Denmark has access to a national health care system that is uniformly organized, tax supported, and provides free access to health care [8]. We used national medical and administrative databases in Denmark to identify the source population of women aged 45–85 years registered in the Civil Registration System (CRS). This registry contains information on civil and vital status for all Danish residents since 1968. Each resident is assigned a unique civil personal registration number (CPR) that permits accurate linkage between registries [9]. 

### Ascertainment of the breast cancer and comparison cohorts

The Danish Cancer Registry (DCR) contains nearly complete data on cancers diagnosed in Denmark [10,11]. Diagnoses were coded according to the *International Classification of Diseases*, revision 7 (ICD-7) until 2003, when recorded diagnoses were converted to ICD-10. From the DCR, we identified female breast cancer patients diagnosed between 1994 and 2008 (ICD-10 code: DC50). We used the CRS to select up to five comparison women from the general population, matched to each breast cancer patient on age and history of the specific comorbidities defined below. The women in the comparison cohort had to be free of breast cancer on the date of breast cancer diagnosis for the corresponding case. The index date was defined as the breast cancer diagnosis date for cases in the breast cancer cohort and also for the women matched to them in the comparison cohort.

#### Comorbidity

The Danish National Registry of Patients (NRP) has recorded all non-psychiatric discharge diagnoses from inpatient admissions since 1977 and from outpatient clinic visits since 1995 [12]. Diagnoses were coded according to ICD-8 1977–1993 and ICD-10 thereafter. The Charlson Comorbidity Index (CCI) provides a summary score based on the presence and severity of 19 individual diseases. It has been validated as a predictor of mortality in breast cancer patients [6]. We used the NRP to identify all recorded diagnoses of diseases, except for breast cancer, included in the CCI for women in the two study cohorts during the ten years before their index date.

#### Mortality

With linkage to the CRS, we followed the breast cancer and matched cohorts until death, emigration or 31 December 2011. Because members of the comparison cohort had no history of breast cancer, we did not ascertain breast-cancer specific or other cause-specific mortality.

#### Statistical analysis

We calculated the frequency of women in the breast cancer cohort and the matched comparison cohort within categories of age (≤50, 51–60, 61–70, 71–80, 81–85 years), year of index date, CCI score (0, 1, 2–3, ≥4), individual diseases included in the CCI index (presence/absence), and, for the breast cancer cohort, cancer stage (local, regional, distant, unknown).

Crude mortality rates with 95% confidence intervals (CIs) were calculated within categories of baseline variables for 0–1 and >1–5 years of follow-up. The matching was dissolved when stratifying the follow-up period, so age-standardized mortality rates were calculated using age weights from the breast cancer cohort on the index date as the standard.

We calculated the interaction contrast (IC), which measures the departure of the mortality rates from an additive model [13]. It is calculated as the difference between the rate differences (mortality rate in the breast cancer cohort minus the mortality rate in the comparison cohort) in the strata with and without comorbidity [13]. We used proportional hazards regression to compute crude hazard ratios as a measure of mortality rate ratios (MRRs), and for the effect of individual diseases, we adjusted for presence of other CCI diseases. For the >1–5 year MRRs, we also adjusted the estimates for age group at diagnosis and year of index date in three categories (1994–1999, 2000–2004, and 2005–2008).

Although chronic pulmonary disease and “any tumor” were prevalent comorbidities in the breast cancer cohort, these diseases did not interact with breast cancer to affect mortality rates. We therefore *a posteriori* repeated all interaction analyses excluding these diseases from the CCI.

The initial cohorts consisted of 48,292 breast cancer patients and 237,938 matched women from the general population. In the breast cancer cohort, 390 (0.81%) women were not matched with any woman in the comparison population. Of these unmatched breast cancer patients, 20% were between 81 and 85 years of age, compared to 9.1% of the matched breast cancer patients. A larger proportion of the unmatched breast cancer patients had a CCI score of ≥4 compared to the matched breast cancer patients (15% vs. 0.9%). Therefore, the combination of old age and multiple comorbidities precluded matching on both age and specific comorbid conditions, resulting in exclusion of these breast cancer patients from the analyses.

Analyses were conducted with SAS version 9.2 (SAS Institute Inc., Cary, NC). This study was approved by the Danish Data Protection Agency (2011-41-6174). No further permissions are needed to conduct studies with no intervention or participant contact in Denmark. 

## Results

Characteristics of the breast cancer and matched cohorts are presented in Table 1. The median age at breast cancer diagnosis was 63.2 years (interquartile range: 55.2 to 73.3 years). The most frequent CCI diseases were cerebrovascular disease (3.7%), chronic pulmonary disease (4.3%), and “any tumor” (3.9%), while hemiplegia (0.1%), leukemia (0.1%), moderate to severe liver disease (0.1%), and AIDS (<0.1%) were among the more rare comorbid diseases. In the breast cancer cohort, 47% had local disease, 40% had regional disease, 6.6% had distant disease, and 7.2% had an unknown breast cancer stage at diagnosis. 

**Table 1:** Characteristics of the breast cancer cohort and the matched population cohort.

	**Breast cancer cohort (n=47,904), Number (%)**	**Matched population cohort (n=237,938), Number (%)**
**Age group (years)**		
≤50	5,085 (11)	25,560 (11)
51-60	13,853 (29)	68,975 (29)
61-70	14,357 (30)	71,193 (30)
71-80	10,262 (21)	50,710 (21)
81-85	4,347 (9.1)	21,500 (9.0)
**Index year**		
1994	2,726 (5.6)	13,564 (5.7)
1995	2,743 (5.7)	13,636 (5.7)
1996	2,890 (6.0)	14,387 (6.1)
1997	2,883 (6.0)	14,356 (6.0)
1998	2,958 (6.2)	14,699 (6.2)
1999	3,087 (6.4)	15,325 (6.4)
2000	3,137 (6.6)	15,601 (6.6)
2001	3,204 (6.7)	15,930 (6.7)
2002	3,407 (7.1)	16,911 (7.1)
2003	3,329 (7.0)	16,504 (6.9)
2004	3,283 (6.8)	16,268 (6.8)
2005	3,279 (6.8)	16,247 (6.8)
2006	3,463 (7.2)	17,162 (7.2)
2007	3,497 (7.2)	17,367 (7.3)
2008	4,018 (8.4)	19,981 (8.4)
**Original CCI score**		
0	38,427 (80.2)	192,135 (81)
1	5303 (11)	26,515 (11.1)
2	2,925 (6.1)	14,432 (6.1)
3	828 (1.7)	3,389 (1.4)
4	205 (0.1)	563 (0.2)
5	25 (0.01)	33 (0.01)
6	167 (0.4)	826 (0.4)
7	23 (0.1)	44 (0.02)
8	1 (0)	1 (0)
**Individual diseases in the CCI**		
Myocardial infarction	680 (1.4)	3124 (1.3)
Congestive heart failure	840 (1.8)	3,724 (1.8)
Peripheral vascular disease	836 (1.8)	3,845 (1.6)
Cerebrovascular disease	1,792 (3.7)	8,479 (3.6)
Dementia	231 (0.5)	1,028 (0.4)
Chronic pulmonary disease	2,054 (4.3)	9,804 (4.1)
Connective tissue disease	934 (2.0)	4,393 (1.9)
Ulcer disease	819 (1.7)	3,808 (2.0)
Mild liver disease	232 (0.5)	1,016 (0.4)
Diabetes I and II	1,229 (2.6)	5,668 (2.0)
Hemiplegia	42 (0.1)	165 (0.1)
Moderate to severe renal disease	209 (0.4)	859 (0.4)
Diabetes with end organ damage	472 (1.0)	2,066 (0.9)
Any tumor (other than breast cancer)	1,856 (3.9)	8,967 (3.8)
Leukemia	43 (0.1)	192 (0.01)
Lymphoma	101 (0.2)	424 (0.2)
Moderate to severe liver disease	39 (0.1)	139 (0.1)
Metastatic solid tumor	188 (0.4)	864 (0.4)
AIDS	1 (0)	5 (0)
**Stage**		
Local	22,338 (47)	
Regional	18,976 (40)	
Distant	3,139 (6.6)	
Unknown	3,451 (7.2)	

### Mortality

Table 2 shows the mortality rates, ICs, and MRRs for 0–1 and >1–5 year mortality in the breast cancer and comparison cohorts. For all CCI score categories, the breast cancer patients had higher mortality rates than the matched cohort. The survival disparities were more marked in the first year of follow-up than in years one to five.

**Table 2:** Mortality rates, adjusted hazard ratios (HRs), and interaction contrasts (ICs) by Charlson Comorbidity Index (CCI) scores for the breast cancer cohort and the matched comparison cohort for 1 year and >1–5 years of follow–up.

	**CCI score**	**No. of deaths**	**Person-years**	**Crude rate (95%CI)/ 1000 person-years^A^**	**IC (95% CI) /1000 person-years**	**Adj HR (95% CI)^B^,^C^**
**0–1 year of follow–up**						
Comparison	0	1,714	191,247	9.0 (8.5, 9.4)		Ref
Breast cancer	0	1,974	37,264	53 (51, 55)	Ref	6.1 (5.7, 6.6)
Comparison	1	1,010	26,021	39 (37, 41)		Ref
Breast cancer	1	500	4,999	100 (92, 109)	17 (7.8, 27)	2.7 (2.4, 3.0)
Comparison	2-3	1,407	17,092	82 (78, 87)		Ref
Breast cancer	2-3	480	3,483	138 (126, 151)	12 (-1.8, 25)	1.6 (1.5, 1.8)
Comparison	≥4	291	1,299	224 (200, 251)		Ref
Breast cancer	≥4	106	357	297 (246, 360)	29 (-33, 91)	1.5 (1.2, 1.9)
**>1–5 years of follow–up**						
Comparison	0	10,411	676,070	18 (17, 19)		Ref
Breast cancer	0	6,244	120,248	57 (54, 60)	Ref	3.6 (3.4, 3.7)
Comparison	1	4,217	83,134	41 (38, 44)		Ref
Breast cancer	1	1,244	14,604	75 (66, 85)	-4.4 (-9.1, 0.4)	1.7 (1.6, 1.9)
Comparison	2-3	3,736	51,098	58 (53, 62)		Ref
Breast cancer	2-3	1,034	9,532	94 (79, 108)	-2.5 (-9.6, 4.1)	1.5 (1.4, 1.6)
Comparison	≥4	403	3,249	111 (86, 136)		Ref
Breast cancer	≥4	124	822	142 (80, 203)	-7.7 (-39, 23)	1.2 (0.9, 1.4)

^A^ Crude rates for 0–1 year of follow–up. For >1–5 years of follow–up, the matching was dissolved and standardized rates were calculated.

^B^ Matching dissolved.

^C^ For >1–5 years of follow–up, HRs were adjusted for age group and index years.

In the first year of follow-up, the interaction between breast cancer and comorbidity accounted for 17 deaths per 1000 person-years (PY) (95% CI: 7.8, 27) for a CCI score of 1, 12 deaths per 1000 PY (95% CI: -1.8, 25) for CCI scores of 2–3, and 29 deaths per 1000 PY (95% CI: -33, 91) for a CCI score ≥4. These represented 17%, 9%, and 10% of total mortality rates, respectively, among the breast cancer patients with comorbid diseases. When the ICs were stratified on breast cancer stage, the interaction observed for the CCI score was primarily driven by distant and unknown stage cancer, as shown in Table 3. The comparison cohort members followed their matched breast cancer patient into the stage category in these stage-stratified analyses. In the 1–5 year survivor cohort, the ICs were near null.

**Table 3:** Interaction contrasts (ICs) and 95% confidence intervals by Charlson comorbidity (CCI) score for 1 year of follow–up.

**Stage**	**Interaction contrast/1000**	**Interaction contrast/1000**	**Interaction contrast/1000**
	**CCI score 1 vs. 0**	**CCI score 1 vs. 0**	**CCI score ≥4 vs. 0**
**Local**	7.8 (-16, 0.53)	-19 (-33, -4.9)	-101 (-168, -35)
**Regional**	0.59 (-11, 12)	-12 (-30, 5.9)	-43 (-125, 39)
**Distant**	228 (115, 341)	150 (28, 272)	370 (11, 729)
**Unknown**	76 (22, 130)	91 (25, 157)	326 (30, 624)

^A^ Crude rates for 0–1 year of follow–up. For >1–5 years of follow–up, the matching was dissolved and standardized rates were calculated.

^B^ Matching dissolved.

^C^ For >1–5 years of follow–up, HRs were adjusted for age group and index years.

Although history at index date of chronic pulmonary disease and “any tumor” were relatively common in the breast cancer cohort, the 0–1 year ICs were only 8.6/1000 PY (95% CI: -8.1, 25) for chronic pulmonary disease and -13/1000 PY (95% CI -31, 5.3) for “any tumor.” When we repeated all analyses for the CCI scores without assigning weights to these two disease types, the 0–1 year overall estimates of the ICs rose from 17 to 21/1000 PY (95% CI: 11, 32) for a CCI score of 1, from 12 to 31/1000 PY (95% CI: 11, 52) for a CCI score of 2–3, and from 29 to 67/1000 PY (95% CI: -19, 152) for a CCI score of ≥4. The ICs for the >1–5 year survivor cohort increased only slightly.

The interaction contrasts between breast cancer and the specific Charlson comorbid diseases were larger during the first year of follow-up than during years one to five of follow-up. The disease with the largest IC in the first year of follow-up was dementia (IC=148/1000PY (95% CI: 58, 239)). When we stratified analyses by breast cancer stage, the interaction between breast cancer and dementia was driven by interaction in the stratum of distant-stage cancers (IC =1150/1000PY (95% CI: 162, 2137)). The ICs for dementia in the strata of local-stage (IC=44/1000PY (95% CI: –68, 155) and regional-stage (IC=-31/1000PY (95% CI: –145, 82) cancers were much smaller. The stage distribution among breast cancer patients with dementia was skewed toward later stage at diagnosis compared with breast cancer patients without dementia. In the first year after breast cancer diagnosis, the mortality rate of breast cancer patients with dementia exceeded that of breast cancer patients without dementia in local-, regional-, and distant-stage strata, yielding a stage-adjusted MRR of 5.0 (95% CI: 3.6, 6.8).

In the first year after diagnosis, there was also interaction between breast cancer and other comorbid diseases, including metastatic solid tumors (IC=66/1000PY, 17% of the total mortality rate), mild liver disease (IC=56/1000PY, 37% of the total mortality rate), moderate to severe renal disease (IC=43/1000PY, 31% of the total mortality rate), and diabetes with end-organ damage (IC=42/1000PY, 27% of the total mortality rate).

**Figure 1:**
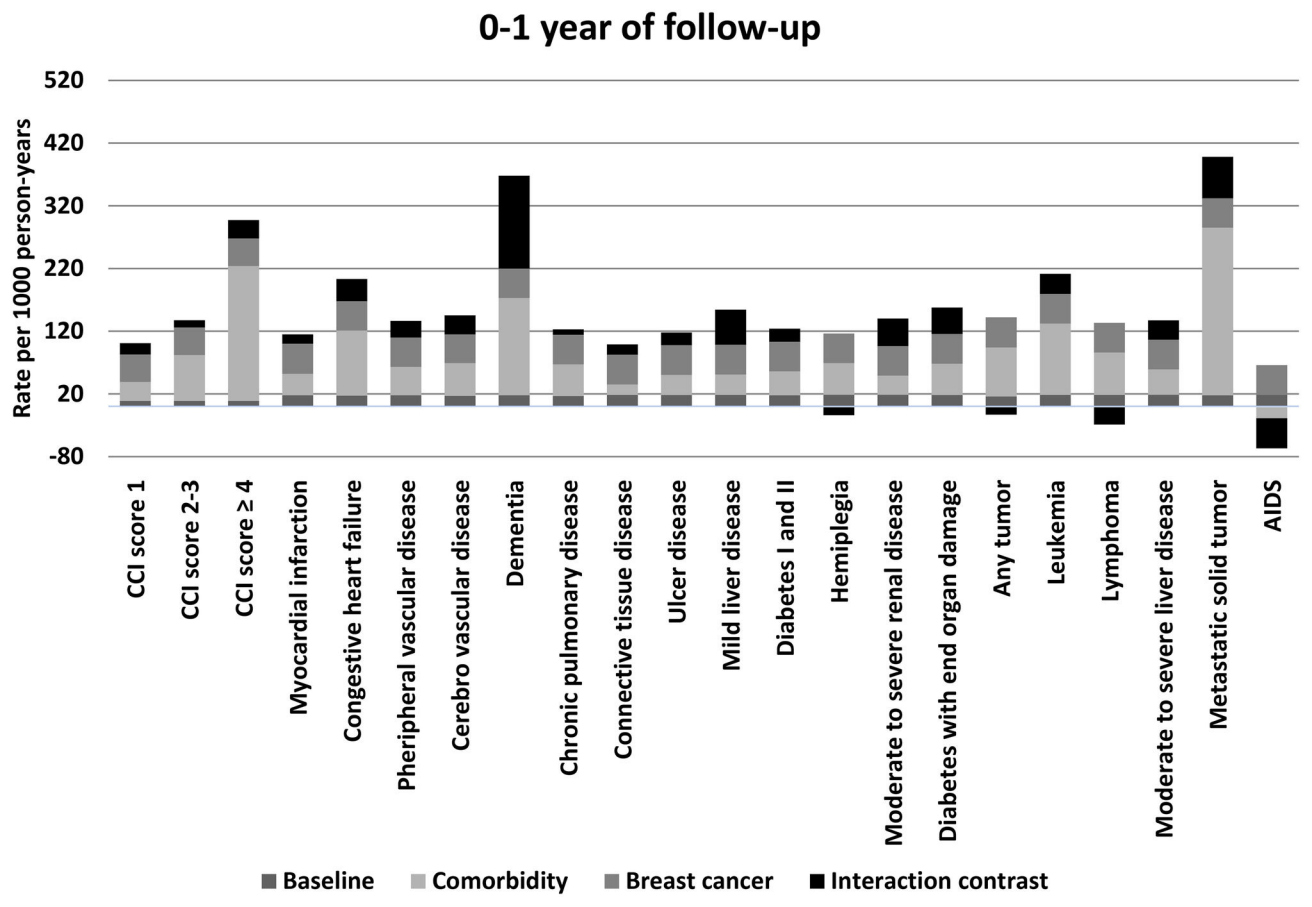
Mortality rates per 1,000 person-years for 0–1 year of follow-up by Charlson Comorbidity Index (CCI) scores and individual diseases in this comorbidity index. The total mortality rate contribution is represented by the baseline rate, comorbidity, breast cancer, and interaction.

**Figure 2:**
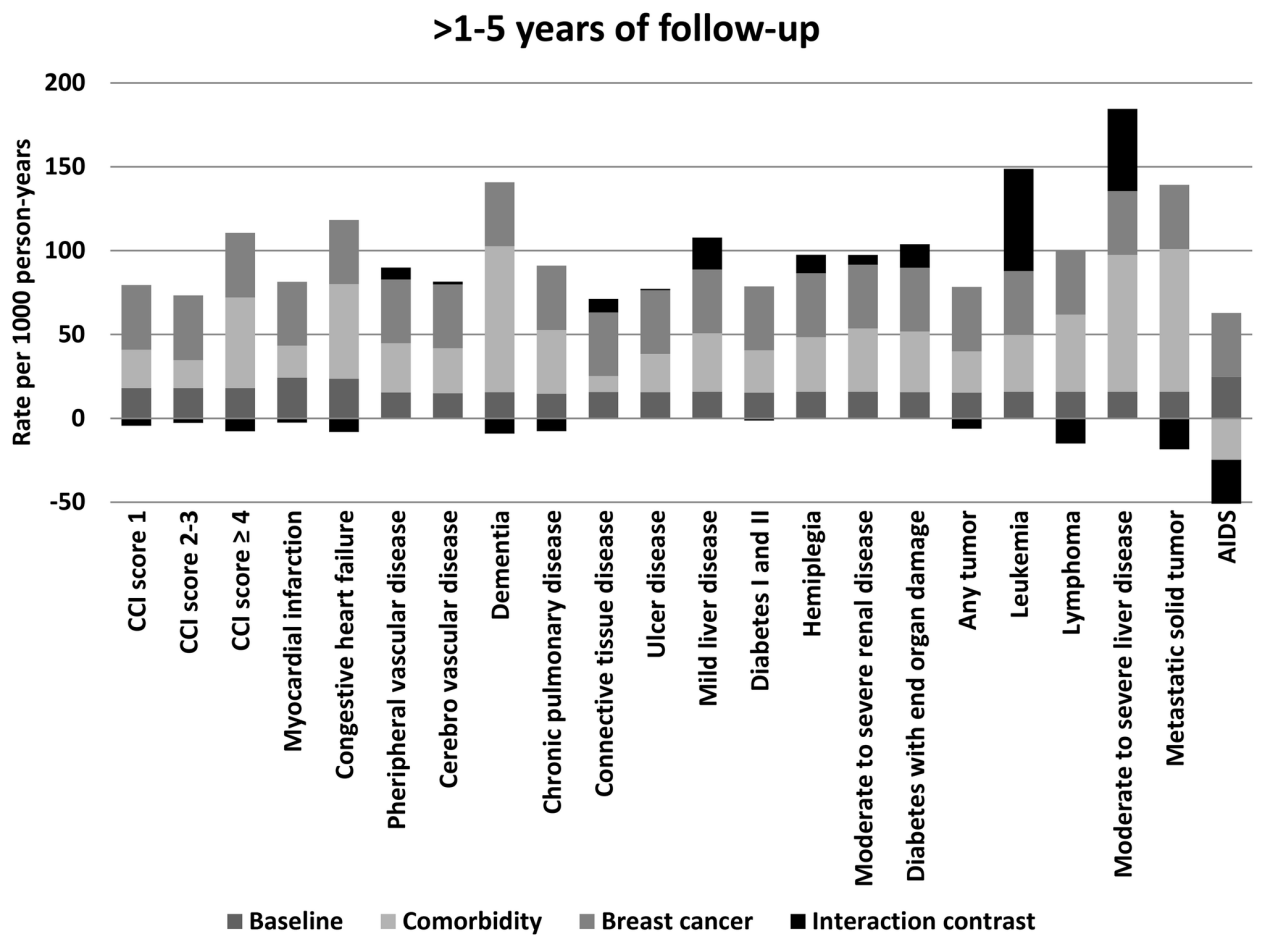
Standardized mortality rates per 1,000 person-years for >1–5 years of follow-up by Charlson Comorbidity Index (CCI) scores and individual diseases in the CCI. The total mortality rate contribution is represented by the baseline rate, comorbidity, breast cancer, and interaction.

In the period one to five years after the index date, there was some interaction between breast cancer and leukemia (IC=61/1000PY, 39% of the total mortality rate), moderate to severe liver disease (IC=49/1000PY, 25% of the total mortality rate), mild liver disease (IC= 19/1000PY, 16% of the total mortality rate), and diabetes with end-organ damage (IC= 14/1000PY, 12% of the total mortality rate). Data for the individual Charlson diseases are presented in Figure 1 and Figure 2 and in Table S1 and Table S2.

## Discussion

In this large, population-based cohort study from Denmark, including more than 47,000 breast cancer patients and 200,000 matched women from the general population, we found that overall mortality in the first year after breast cancer diagnosis was influenced by interaction between breast cancer and comorbid diseases present at diagnosis. The interaction was most pronounced in the strata of distant and unknown breast cancer stage. Among individual diseases, dementia interacted most strongly with breast cancer, but metastatic solid tumors, mild liver disease, moderate to severe renal disease, and diabetes with end-organ damage also showed strong interactions. In the >1–5 year survivor cohort, there was no strong interaction with the CCI summary comorbidity score, although some interaction was observed with leukemia, moderate to severe liver disease, mild liver disease, and diabetes with end-organ damage. 

A particular strength of this study is the inclusion of a comparison cohort free of breast cancer matched to the breast cancer cohort on specific comorbidities, which allows for the study of disease-specific clinical interactions between breast cancer and comorbidity. A concomitant limitation was our inability to study disease-specific causes of death, since members of the comparison cohort were unlikely to die of breast cancer. We included all women with breast cancer diagnoses from the entire country and achieved complete follow-up through the CRS. Registration of breast cancer in the DCR is nearly complete [14]. The validity of the CCI diseases recorded in the NRP has been shown to be consistently high [15]. However, outpatient data were not included before 1995, so under-registration could bias results. Such misclassification should bias the comparison of mortality in breast cancer patients with mortality in the comparison cohort toward the null, since the misclassification rate should not depend on the subsequent breast cancer diagnosis. The impact of misclassification on estimates of the interaction contrast is less predictable [16]. In addition, we lacked information on potential other confounders, such as lifestyle-related factors.

The interaction between breast cancer and comorbidity was mainly observed during the first year after breast cancer diagnosis, possibly due to lack of focus on care for comorbid diseases during cancer treatment. A recent study based on SEER data showed equal quality of care for comorbid conditions in breast cancer patients and non-cancer controls, but this was at three years after the cancer diagnosis [17]. In the time period one to five years after breast cancer diagnosis, we observed no substantial interaction between breast cancer and comorbid diseases, possibly due to equal quality of care of comorbid conditions in the period after completion of primary breast cancer treatment.

Interaction contrasts were negative in some analyses, although often imprecisely measured. Negative interaction contrasts were observed most often in the local and regional stage categories. This pattern suggests that prevalent and well-managed comorbidities brought breast cancer patients to medical attention and diagnosis sooner, resulting in a stage-shift to earlier and more treatable breast cancers within the early-stage categories. In later stage categories, breast cancer patients with severe comorbidity may be diagnosed with breast cancer late and at an unfavorable cancer stage, as some comorbid conditions could mask evidence of this cancer [18]. We have clearly demonstrated this explanation for breast cancer patients with a CCI score ≥4 and for patients with dementia. Breast cancer patients with severe comorbidity may not receive cancer treatment in accordance with the treatment guidelines [19,20], because the comorbidity, its treatment, the cancer treatment, or its side-effects preclude the most aggressive treatments. Less aggressive treatment of cancer patients with dementia has been previously documented [21-23], which provides one explanation for the excess mortality rate for breast cancer patients with dementia in the first year after their breast cancer diagnosis.

To our knowledge, this study is the first to report specific interaction contrasts between breast cancer and the CCI score or individual diseases included in the CCI that affect the mortality rate. Studies that lacked a cohort free of breast cancer have shown that comorbidity and associated suboptimal breast cancer treatment increase the risk of death without recurrence in older women [2]. Other studies did not report increased mortality due to causes other than breast cancer in breast cancer cohorts compared to the general population [24,25]. However, a Swedish study reported increased mortality associated with diseases of the heart, pulmonary circulation (pulmonary embolism and other diseases of pulmonary vessels), and gastric diseases [26]. In addition to the interaction with dementia, we also observed interactions between breast cancer and renal diseases, liver diseases, diabetes, and other cancers. Compared to breast cancer patients without these comorbid diseases, patients with these comorbidities may not tolerate adjuvant chemotherapy and radiotherapy as well [27,28].

In summary, our study shows a clinical interaction between prevalent comorbidities and overall mortality in breast cancer patients—particularly within one year after breast cancer diagnosis and mainly in patients with distant and unknown stage breast cancer. There was substantial interaction between dementia and breast cancer, suggesting that these patients tend to have breast cancer diagnosed at later stages. Successful treatment of the comorbid diseases or the breast cancer can delay mortality caused by this interaction. 

## Supporting Information

Table S1 **Crude mortality rates, adjusted HRs, and interaction contrasts (ICs) by individual diseases in the Charlson Comorbidity Index for the breast cancer cohort and the matched comparison cohort during 0-1 year of follow–up.**
(DOCX)

Table S2 **Standardized mortality rates, adjusted HRs, and interaction contrasts (ICs) by individual diseases in the Charlson Comorbidity Index for the breast cancer cohort and the matched comparison cohort during 1-5 years of follow–up.**
(DOCX)
