# Supplementary material for: Comorbid Diseases Interact with Breast Cancer to Affect Mortality in the First Year after Diagnosis—A Danish Nationwide Matched Cohort Study
Source: PLoS One. 2013 Oct 9;8(10):e76013. doi: 10.1371/journal.pone.0076013 (PMC3794020; doi:10.1371/journal.pone.0076013)
Supplement: Table S1 — Crude mortality rates, adjusted HRs, and interaction contrasts (ICs) by individual diseases in the Charlson Comorbidity Index for the breast cancer cohort and the matched comparison cohort during 0-1 year of follow–up. (DOCX) [file pone.0076013.s001.docx]

| **Table S1. Crude mortality rates, adjusted HRs, and interaction contrasts (ICs) by individual diseases in the Charlson Comorbidity Index for the breast cancer cohort and the matched comparison cohort during 0-1 year of follow–up.** | | | | | | |
| --- | --- | --- | --- | --- | --- | --- |
|  | **Presence of disease** | **No. of deaths** | **Person-years** | **Crude rate (95% CI)/ 1000 person-years** | **IC (95% CI)/**  **1000 person-years** | **Adj HR (95% CI) ^A^** |
| **Myocardial infarction** | | |  |  |  |  |
| Comparison | No | 4,264 | 232,620 | 18.3 (17.8, 18.9) |  | Ref |
| Breast cancer | No | 2987 | 45,463 | 65.7 (63.4, 68.1) | Ref | 3.4 (3.3, 3.6) |
| Comparison | Yes | 158 | 3,039 | 52.0 (44.5, 60.8) |  | Ref |
| Breast cancer | Yes | 73 | 6389 | 114 (90.9, 144) | 15 (-13, 43) | 2.0 (1.5, 2.6) |
| **Congestive heart failure** | | |  |  |  |  |
| Comparison | No | 3,999 | 232,156 | 17.2 (16.7, 17.8) |  | Ref |
| Breast cancer | No | 2,907 | 45,348 | 64.1 (61.8, 66.5) | Ref | 3.6 (3.4, 3.8) |
| Comparison | Yes | 423 | 3,503 | 121 (110, 133) |  | Ref |
| Breast cancer | Yes | 153 | 754 | 203 (173, 238) | 35 (1.1, 70) | 1.6 (1.3, 1.9) |
| **Peripheral vascular disease** | | |  |  |  |  |
| Comparison | No | 4,189 | 231,932 | 18.1 (17.5, 18.6) |  | Ref |
| Breast cancer | No | 2,955 | 45,331 | 65.2 (62.9, 67.6) | Ref | 3.5 (3.3, 3.7) |
| Comparison | Yes | 233 | 3,726 | 62.5 (55.0, 71.1) |  | Ref |
| Breast cancer | Yes | 105 | 771 | 136 (113, 165) | 27 (-0.84, 54) | 1.9 (1.5, 2.5) |
| **Cerebrovascular disease** | | |  |  |  |  |
| Comparison | No | 3,860 | 227,460 | 17.0 (16.4, 17.5) |  | Ref |
| Breast cancer | No | 2,820 | 44,452 | 63.4 (61.1, 65.8) | Ref | 3.6 (3.5, 3.8) |
| Comparison | Yes | 562 | 8,198 | 68.5 (63.1, 74.5) |  | Ref |
| Breast cancer | Yes | 240 | 1,650 | 145 (128, 165) | 30 (11, 50) | 2.0 (1.7, 2.3) |
| **Dementia** | |  |  |  |  |  |
| Comparison | No | 4,258 | 234,713 | 18.1 (17.6, 18.7) |  | Ref |
| Breast cancer | No | 2,990 | 45,912 | 65.1 (62.8, 67.5) | Ref | 3.5 (3.3, 3.6) |
| Comparison | Yes | 164 | 946 | 173 (149, 202) |  | Ref |
| Breast cancer | Yes | 70 | 190 | 369 (292, 466) | 148 (58, 239) | 2.0 (1.5, 2.7) |
| **Chronic pulmonary disease** | | |  |  |  |  |
| Comparison | No | 3,788 | 226,174 | 16.7 (16.2, 17.3) |  | Ref |
| Breast cancer | No | 2,825 | 44,186 | 63.9 (61.6, 66.3) | Ref | 3.7 (3.5, 3.8) |
| Comparison | Yes | 634 | 9,484 | 66.9 (61.8, 72.3) |  | Ref |
| Breast cancer | Yes | 235 | 1,916 | 123 (108, 139) | 8.6 (-8.1, 25) | 1.7 (1.5, 2.0) |
| **Connective tissue disease** | | |  |  |  |  |
| Comparison | No | 4,272 | 231,342 | 18.5 (17.9, 19.0) |  | Ref |
| Breast cancer | No | 2,973 | 45,218 | 65.7 (63.4, 68.2) | Ref | 3.4 (3.3, 3.6) |
| Comparison | Yes | 150 | 4,316 | 34.8 (29.6, 40.8) |  | Ref |
| Breast cancer | Yes | 87 | 884 | 98.4 (79.8, 121) | 16 (-5.2, 38) | 2.5 (1.9, 3.3) |
| **Ulcer disease** | |  |  |  |  |  |
| Comparison | No | 4,236 | 231,943 | 18.3 (17.7, 18.8) |  | Ref |
| Breast cancer | No | 2,970 | 45,336 | 65.5 (63.2, 67.9) | Ref | 3.5 (3.3, 3.6) |
| Comparison | Yes | 186 | 3,715 | 50.1 (43.4, 57.8) |  | Ref |
| Breast cancer | Yes | 90 | 766 | 118 (95.6, 145) | 20 (-5.2, 46) | 2.1 (1.6, 2.7) |
| **Mild liver disease** | |  |  |  |  |  |
| Comparison | No | 4,371 | 234,666 | 18.6 (18.1, 19.2) |  | Ref |
| Breast cancer | No | 3,027 | 45,889 | 66.0 (63.6, 68.4) | Ref | 3.4 (3.2, 3.6) |
| Comparison | Yes | 51 | 993 | 51.4 (39.1, 67.6) |  | Ref |
| Breast cancer | Yes | 33 | 213 | 155 (110, 218) | 56 (1.6, 111) | 2.8 (1.8, 4.4) |
| **Diabetes I and II** | |  |  |  |  |  |
| Comparison | No | 4,114 | 230,146 | 17.9 (17.3, 18.4) |  | Ref |
| Breast cancer | No | 2,918 | 44,953 | 65 (63, 67) | Ref | 3.5 (3.3, 3.7) |
| Comparison | Yes | 308 | 5,512 | 55.9 (50.0, 62.5) |  | Ref |
| Breast cancer | Yes | 142 | 1,149 | 124 (105, 146) | 21 (-0.7, 42) | 2.0 (1.7, 2.5) |
| **Hemiplegia** | |  |  |  |  |  |
| Comparison | No | 4,411 | 235,498 | 18.7 (18.2, 19.3) |  | Ref |
| Breast cancer | No | 3,056 | 46,063 | 66.3 (64.0, 68.7) | Ref | 3.4 (3.2, 3.6) |
| Comparison | Yes | 11 | 160 | 68.6 (38.0, 124) |  | Ref |
| Breast cancer | Yes | 4 | 39 | 102 (38.5, 273) | -14 (-122, 95) | 1.4 (0.4, 4.3) |
| **Moderate to severe renal disease** | | | |  |  |  |
| Comparison | No | 4,381 | 234,822 | 18.7 (18.1, 19.2) |  | Ref |
| Breast cancer | No | 3,033 | 45,909 | 66.1 (63.8, 68.5) | Ref | 3.4 (3.2, 3.6) |
| Comparison | Yes | 41 | 837 | 49.0 (36.1, 66.6) |  | Ref |
| Breast cancer | Yes | 27 | 193 | 140 (95.9, 204) | 43 (-12, 98) | 2.3 (1.4, 3.9) |
| **Diabetes with end organ damage** | | | |  |  |  |
| Comparison | No | 4,287 | 233,659 | 18.3 (17.8, 18.9) |  | Ref |
| Breast cancer | No | 2,992 | 45,667 | 65.5 (63.2, 67.9) | Ref | 3.4 (3.3, 3.6) |
| Comparison | Yes | 135 | 2,000 | 67.5 (57.0, 79.9) |  | Ref |
| Breast cancer | Yes | 68 | 435 | 156 (123, 198) | 42 (2.7, 81) | 2.2 (1.6, 3.0) |
| **Any tumor** | |  |  |  |  |  |
| Comparison | No | 3,618 | 227,134 | 15.9 (15.4, 16.5) |  | Ref |
| Breast cancer | No | 2,836 | 44,370 | 63.9 (61.6, 66.3) | Ref | 3.8 (3.7, 4.1) |
| Comparison | Yes | 804 | 8,525 | 94.3 (88.0, 101) |  | Ref |
| Breast cancer | Yes | 224 | 1,732 | 129 (113, 147) | -13 (-31, 5.3) | 1.3 (1.1, 1.5) |
| **Leukemia** | |  |  |  |  |  |
| Comparison | No | 4,398 | 235,476 | 18.7 (18.1, 19.2) |  | Ref |
| Breast cancer | No | 3,052 | 46,064 | 66.3 (63.9, 68.6) | Ref | 3.4 (3.2, 3.6) |
| Comparison | Yes | 24 | 183 | 132 (88.2, 196) |  | Ref |
| Breast cancer | Yes | 8 | 38 | 211 (105, 421) | 32 (-124, 187) | 1.6 (0.7, 3.6) |
| **Lymphoma** | |  |  |  |  |  |
| Comparison | No | 4,387 | 235,252 | 18.6 (18.1, 19.2) |  | Ref |
| Breast cancer | No | 3,050 | 46,007 | 66.3 (63.9, 68.7) | Ref | 3.4 (3.3, 3.6) |
| Comparison | Yes | 35 | 406 | 86.2 (61.9, 120) |  | Ref |
| Breast cancer | Yes | 10 | 95 | 105 (55.6, 195) | -29 (-100, 43) | 0.9 (0.4, 1.9) |
| **Moderate to severe liver disease** | | | |  |  |  |
| Comparison | No | 4,414 | 235,523 | 18.7 (18.2, 19.3) |  | Ref |
| Breast cancer | No | 3,055 | 46,066 | 66.3 (64.0, 68.7) | Ref | 3.4 (3.2, 3.6) |
| Comparison | Yes | 8 | 135 | 59.3 (29.7, 119) |  | Ref |
| Breast cancer | Yes | 5 | 36 | 138 (57.6, 332) | 31 (-97, 159) | 2.6 (0.9, 8.1) |
| **Metastatic solid tumor** | | |  |  |  |  |
| Comparison | No | 4,211 | 234,917 | 17.9 (17.4, 18.5) |  | Ref |
| Breast cancer | No | 3,001 | 45,954 | 65.3 (63.0, 67.7) | Ref | 3.5 (3.3, 3.7) |
| Comparison | Yes | 211 | 741 | 285 (249, 326) |  | Ref |
| Breast cancer | Yes | 59 | 148 | 398 (309, 514) | 66 (-43, 175) | 1.3 (1.0, 1.8) |
| **AIDS** |  |  |  |  |  |  |
| Comparison | No | 4,422 | 235,653 | 18.8 (18.2, 19.3) |  | Ref |
| Breast cancer | No | 3,060 | 46,101 | 66.4 (64.1, 68.8) | Ref | 3.4 (3.2, 3.5) |
| Comparison | Yes | 0 | 5 | - |  | Ref |
| Breast cancer | Yes | 0 | 1 | - | -48 (-50, -45) | - |
| ^A^ Matching dissolved. Diseases are adjusted for the other diseases in the CCI. | | | | | | |
