# Supplementary material for: Comorbid Diseases Interact with Breast Cancer to Affect Mortality in the First Year after Diagnosis—A Danish Nationwide Matched Cohort Study
Source: PLoS One. 2013 Oct 9;8(10):e76013. doi: 10.1371/journal.pone.0076013 (PMC3794020; doi:10.1371/journal.pone.0076013)
Supplement: Table S2 — Standardized mortality rates, adjusted HRs, and interaction contrasts (ICs) by individual diseases in the Charlson Comorbidity Index for the breast cancer cohort and the matched comparison cohort during 1-5 years of follow–up. (DOCX) [file pone.0076013.s002.docx]

| **Table S2. Standardized mortality rates, adjusted HRs, and interaction contrasts (ICs) by individual diseases in the Charlson Comorbidity Index for the breast cancer cohort and the matched comparison cohort during 1-5 years of follow–up.** | | | | | | | |
| --- | --- | --- | --- | --- | --- | --- | --- |
|  | **Presence of disease** | | **No. of deaths** | **Person-years** | **Adj rate(95%CI)/ 1000 person-years^A^** | **IC (95%CI)/**  **1000 person-years ^A^** | **Adj HR (95%CI)^A,B^** |
| **Myocardial infarction** | | | |  |  |  |  |
| Comparison | | No | 18,134 | 803,881 | 24.3 (23.6, 25.1) |  | Ref |
| Breast cancer | | No | 8,452 | 143,392 | 62.4 (59.5, 65.3) | Ref | 2.67 (2.60, 2.74) |
| Comparison | | Yes | 633 | 9,669 | 43.3 (34.4, 52.0) |  | Ref |
| Breast cancer | | Yes | 194 | 1,813 | 78.8 (54.5, 103) | -2.5 (-16, 11) | 1.6 (1.4, 1.9) |
| **Congestive heart failure** | | | |  |  |  |  |
| Comparison | | No | 17,550 | 804,082 | 23.6 (22.9, 24.4) |  | Ref |
| Breast cancer | | No | 8,357 | 143,343 | 61.9 (59.0, 64.7) | Ref | 2.75 (2.68, 2.82) |
| Comparison | | Yes | 1,217 | 9,468 | 80.0 (64.4, 95.6) |  | Ref |
| Breast cancer | | Yes | 289 | 1,862 | 110 (64.3, 156) | -8.1 (-32, 16) | 1.1 (1.0, 1.3) |
| **Pheripheral vascular disease** | | | |  |  |  |  |
| Comparison | | No | 17,931 | 802,273 | 24.1 (23.4, 24.8) |  | Ref |
| Breast cancer | | No | 8398 | 143,158 | 62.1 (59.2, 65.0) | Ref | 2.68 (2.61, 2.75) |
| Comparison | | Yes | 836 | 11,277 | 53.4 (44.6, 62.2) |  | Ref |
| Breast cancer | | Yes | 248 | 2,047 | 98.5 (67.6, 129) | 7.1 (-7.6, 22) | 1.6 (1.4, 1.8) |
| **Cerebro vascular disease** | | | |  |  |  |  |
| Comparison | | No | 16,907 | 789,032 | 23.4 (22.7, 24.1) |  | Ref |
| Breast cancer | | No | 8,133 | 140,780 | 61.5 (58.6, 64.4) | Ref | 2.76 (2.69, 2.84) |
| Comparison | | Yes | 1,860 | 24,518 | 50.3 (44.5, 56.2) |  | Ref |
| Breast cancer | | Yes | 513 | 4,425 | 90.0 (69.7, 110) | 1.6 (-8.1, 11) | 1.50 (1.3, 1.6) |
| **Dementia** | | |  |  |  |  |  |
| Comparison | | No | 18,305 | 811,410 | 24.2 (23.5, 24.9) |  | Ref |
| Breast cancer | | No | 8,556 | 144,828 | 62.4 (59.5, 65.3) | Ref | 2.67 (2.60, 2.74) |
| Comparison | | Yes | 462 | 2,140 | 111 (80.2, 142) |  | Ref |
| Breast cancer | | Yes | 90 | 377 | 140 (52.6, 228) | -9.1 (-60, 42) | 1.1 (0.9, 1.3) |
| **Chronic pulmonary disease** | | | |  |  |  |  |
| Comparison | | No | 16,843 | 785,091 | 23.1 (22.4, 23.8) |  | Ref |
| Breast cancer | | No | 8,126 | 139,879 | 61.5 (58.6, 64.4) | Ref | 2.78 (2.71, 2.86) |
| Comparison | | Yes | 1,924 | 28,460 | 61.0 (55.1, 66.8) |  | Ref |
| Breast cancer | | Yes | 520 | 5,326 | 91.8 (73.9, 110) | -7.6 (-16, 1.2) | 1.4 (1.3, 1.5) |
| **Connective tissue disease** | | | |  |  |  |  |
| Comparison | | No | 18,185 | 799,598.2 | 24.5 (23.7, 25.2) |  | Ref |
| Breast cancer | | No | 8,423 | 142,674.6 | 62.4 (59.5, 65.3) | Ref | 2.64 (2.58, 2.71) |
| Comparison | | Yes | 582 | 13,952.1 | 34.0 (28.2, 39.9) |  | Ref |
| Breast cancer | | Yes | 223 | 2,530.5 | 80.1 (56.1, 104) | 8.1 (-3.2, 19) | 2.0 (1.8, 2.4) |
| **Ulcer disease** | | |  |  |  |  |  |
| Comparison | | No | 18,054 | 801,715.2 | 24.3 (23.6, 25.0) |  | Ref |
| Breast cancer | | No | 8,417 | 143,051 | 62.2 (59.3, 65.1) | Ref | 2.67 (2.60, 2.74) |
| Comparison | | Yes | 713 | 11835 | 47.2 (38.7, 55.7) |  | Ref |
| Breast cancer | | Yes | 229 | 2,155 | 85.9 (60.0, 112) | 0.75 (-12, 13) | 1.7 (1.5, 2.0) |
| **Mild liver disease** | | |  |  |  |  |  |
| Comparison | | No | 18,606 | 810,516 | 24.5 (23.8, 25.3) |  | Ref |
| Breast cancer | | No | 8,583 | 144,631 | 62.6 (60.0, 65.5) | Ref | 2.63 (2.57, 2.70) |
| Comparison | | Yes | 161 | 3,035 | 59.4 (36.2, 82.6) |  | Ref |
| Breast cancer | | Yes | 63 | 575 | 117 (45.3, 188) | 19 (-16, 55) | 1.9 (1.4, 2.6) |
| **Diabetes I and II** | | |  |  |  |  |  |
| Comparison | | No | 17,683 | 796,599 | 24.0 (23.2, 24.7) |  | Ref |
| Breast cancer | | No | 8,318 | 141,982 | 62.1 (59.1, 65.0) | Ref | 2.7 (2.6, 2.8) |
| Comparison | | Yes | 1,084 | 16,951 | 49.3 (42.9, 55.6) |  | Ref |
| Breast cancer | | Yes | 328 | 3,223 | 86.0 (63.8, 108) | -1.3 (-12, 9.3) | 1.5 (1.3, 1.7) |
| **Hemiplegia** | | |  |  |  |  |  |
| Comparison | | No | 18,734 | 813,061.2 | 24.7 (23.9, 25.4) |  | Ref |
| Breast cancer | | No | 8,633 | 145,098.6 | 62.8 (59.9, 65.6) | Ref | 2.63 (2.56, 2.70) |
| Comparison | | Yes | 33 | 489.2 | 57.2 (17.8, 96.7) |  | Ref |
| Breast cancer | | Yes | 13 | 106.5 | 106 (-8.48, 221) | 11 (-53, 75) | 1.5 (0.77, 3.0) |
| **Moderate to severe renal disease** | | | | |  |  |  |
| Comparison | | No | 18,607 | 811,031.7 | 24.6 (23.8, 25.3) |  | Ref |
| Breast cancer | | No | 8,589 | 144,692 | 62.6 (59.7, 65.5) |  | 2.64 (2.57, 2.71) |
| Comparison | | Yes | 160 | 2,519 | 62.4 (41.9, 82.8) | Ref | Ref |
| Breast cancer | | Yes | 57 | 513 | 106 (47.9, 165) | 5.8 (-24, 35) | 1.4 (1.04, 2.0) |
| **Diabetes with end organ damage** | | | | |  |  |  |
| Comparison | | No | 18,308 | 807,651 | 24.3 (23.6, 25.0) |  | Ref |
| Breast cancer | | No | 8,505 | 144,054 | 62.4 (59.5, 65.3) | Ref | 2.66 (2.59, 2.73) |
| Comparison | | Yes | 459 | 5,900 | 60.4 (47.3, 73.6) |  | Ref |
| Breast cancer | | Yes | 141 | 1,151 | 113 (63.3, 162) | 14 (-10, 38) | 1.5 (1.2, 1.8) |
| **Any tumor** | | |  |  |  |  |  |
| Comparison | | No | 17,292 | 787,377 | 23.9 (23.1, 24.6) |  | Ref |
| Breast cancer | | No | 8,214 | 140,246 | 62.3 (59.3, 65.2) | Ref | 2.73 (2.66, 2.81) |
| Comparison | | Yes | 1,475 | 26,173 | 48.5 (42.7, 54.4) |  | Ref |
| Breast cancer | | Yes | 432 | 4,959 | 80.8 (62.0, 99.5) | -6.2 (-15, 2.8) | 1.5 (1.3, 1.7) |
| **Leukemia** | | |  |  |  |  |  |
| Comparison | | No | 18,730 | 813,040 | 24.7 (23.9, 25.4) |  | Ref |
| Breast cancer | | No | 8,632 | 145,121 | 62.7 (59.8, 65.6) | Ref | 2.63 (2.56, 2.69) |
| Comparison | | Yes | 37 | 510 | 58.6 (18.4, 98.8) |  | Ref |
| Breast cancer | | Yes | 14 | 85 | 157 (-6.76, 321) | 61 (-30, 151) | 2.4 (1.3, 4.6) |
| **Lymphoma** | | |  |  |  |  |  |
| Comparison | | No | 18,670 | 812,306 | 24.6 (23.9, 25.3) |  | Ref |
| Breast cancer | | No | 8,618 | 144,935 | 62.7 (59.8, 65.6) | Ref | 2.63 (2.57, 2.70) |
| Comparison | | Yes | 97 | 1245 | 70.5 (39.4, 102) |  | Ref |
| Breast cancer | | Yes | 28 | 270 | 94.2 (24.4, 164) | -15 (-53, 24) | 1.2 (0.8, 1.9) |
| **Moderate to severe liver disease** | | | | |  |  |  |
| Comparison | | No | 18,736 | 813,165 | 24.6 (23.9, 25.4) |  | Ref |
| Breast cancer | | No | 8,633 | 145,120 | 62.7 (59.8, 65.6) | Ref | 2.63 (2.56, 2.70) |
| Comparison | | Yes | 31 | 385 | 106 (-8.79, 221) |  | Ref |
| Breast cancer | | Yes | 13 | 86 | 193 (-59.3, 446) | 49 (-95, 193) | 1.65 (0.83, 3.27) |
| **Metastatic solid tumor** | | | |  |  |  |  |
| Comparison | | No | 18,565 | 811,748.8 | 24.5 (23.8, 25.2) |  | Ref |
| Breast cancer | | No | 8,605 | 144,849.5 | 62.7 (59.8, 65.5) | Ref | 2.65 (2.58, 2.72) |
| Comparison | | Yes | 202 | 1,801.6 | 110 (76.4, 143) |  | Ref |
| Breast cancer | | Yes | 41 | 355.7 | 129 (36.3, 223) | -19 (-64, 27) | 0.94 (0.70, 1.3) |
| **AIDS** | |  |  |  |  |  |  |
| Comparison | | No | 18,767 | 813,530.4 | 24.7 (24.0, 25.4) |  | Ref |
| Breast cancer | | No | 8,646 | 145,201.2 | 62.8 (59.9, 65.7) | Ref | 2.63 (2.56, 2.70) |
| Comparison | | Yes | 0 | 20.0 | 0.0 |  | Ref |
| Breast cancer | | Yes | 0 | 4.0 | 0.0 | -38 (-39, -37) | - |
| ^A^ Matching dissolved. ^B^ HRs are adjusted for other diseases in the CCI. | | | | | | | |
